# Molecular Dynamics Simulations of Supercooled Aqueous Solutions of Calcium Perchlorate: Thermodynamics and Structure of Martian Solutes in TIP4P/2005 Water

**DOI:** 10.1021/acs.jpcb.5c03712

**Published:** 2025-08-14

**Authors:** Paolo La Francesca, Paola Gallo

**Affiliations:** † Dipartimento di Matematica e Fisica, Università degli studi Roma Tre, via della Vasca Navale 84, 00146 Roma, Italy; ‡ Network Science Institute, Department of Physics, Northeastern University, Boston, Massachusetts 02115, United States

## Abstract

We employ molecular dynamics simulations to determine how calcium perchlorate modifies the phase diagram and structure of supercooled TIP4P/2005 water. These solutions are particularly relevant in light of recent experimental evidence of liquid water in perchlorate solutions underneath Martian soil. We focus on the interplay between its low-density liquid (LDL) and high-density liquid (HDL) phases, simulating solutions at concentrations of 3.15 and 25.7 wt%. Thermodynamic analysis confirms the persistence of several water anomalies, albeit shifted by the solutes. A second-order liquid–liquid critical point (LLCP) is observed at a concentration of 3.15 wt%. Structurally, radial distribution functions demonstrate that upon increasing the solute concentration, the HDL-like behavior is enhanced, as the thermodynamic LDL-like region appears to be shrinking. Calcium ions with perchlorate ions in water appear to be particularly effective at favoring supercooling.

## Graphical abstract

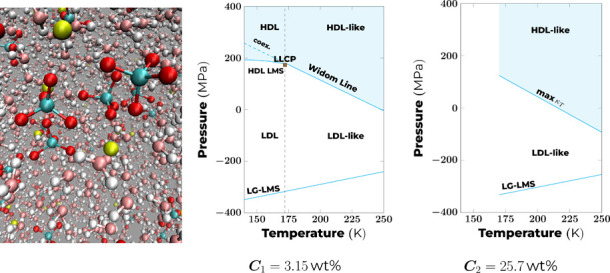

## 1 Introduction

Water, one of the most common and vital substances on Earth, displays a variety of anomalous physical properties that make it unique, compared to other liquids.^1–6^ These anomalies persist when water is supercooled, i.e., when kept liquid below its freezing temperature, resulting in apparent singularities, the explanation for which is an important subject of investigation.

The experimental study of supercooled water is subject to the challenges arising from nucleation, which naturally occurs in systems undergoing first-order phase transitions, the water-to-ice transition being one of them. From a thermodynamic point of view, indeed, there are no inherent obstacles preventing supercooled water to reach a glassy phase through a reversible path.^7^ However, the natural presence of impurities in water significantly enhances the nucleation process, making supercooled water more likely to crystallize. This is related to the local tendency of water to form a distorted tetrahedron with the nearest neighbors, from which ice is easily formed.^2^ Moreover, in the supercooled region known as no man’s land, nucleation occurs so quickly in pure water that the characteristic timescales for observing supercooled water are exceeded, making experimental access to this region particularly difficult.^8^

One of the most credited hypotheses to explain the anomalous behavior of water is the existence of a second-order liquid–liquid critical point (LLCP) in its supercooled region. This hypothesis, first proposed by Poole, Sciortino, Essman, and Stanley in 1992 ^9^ based on Molecular Dynamics (MD) simulations on the ST2 water model,^10^ suggests that below the liquid–liquid critical temperature, water can exist in two distinct phases: high-density liquid (HDL) and low-density liquid (LDL). Simulations have rigorously confirmed the existence of the LLCP in several classic computational models for water, including ST2 (see refs 11–16 ), Jagla^17^ (see ref 18), TIP4P/2005,^19^ and TIP4P/Ice^20^ (see ref 21 for both). First-principle-derived water models such as WAIL,^22,23^ rWAIL,^24^ and MB-Pol^25^ also show the existence of an LLCP. Although directly corroborating the liquid–liquid phase transition theory through experiments remains challenging due to the extremely rapid nucleation in the no man’s land, where the LLCP is believed to reside, strong indirect experimental evidence supports its existence.^26–30^

Important recent advancements have narrowed the experimental boundaries of the no man’s land.^27,31,32^ On the other hand, simulations,^6,33^ because of the fast cooling rates they allow to be implemented, remain a crucial means to explore the whole phase diagram of supercooled water, including regions that are still experimentally inaccessible.

Through MD simulations, in particular, it was proven that the TIP4P/2005, one of the most accurate models available for reproducing the phase diagram of water, exhibits a second-order Ising-like liquid–liquid phase transition at *T*_C_ = 172 K and *p*_C_ = 186 MPa.^21^ According to the theory of critical phenomena, as a second-order critical point is approached, a line of state points corresponding to the correlation length maxima, known as the Widom line, emerges.^34^ This line separates, in the one-phase region, two portions of the phase diagram in which HDL or LDL fluctuations in time prevail over those of the other component. Both simulations and experiments have confirmed the existence of the Widom line in the supercooled region of the phase diagram of water, further supporting the LLCP hypothesis.^27,34–39^

The study of water anomalies is closely linked to the investigation of supercooled aqueous solutions, as water is naturally found and often easier to supercool in them. Besides, simulations have shown that the LLCP and other anomalies of water persist, in solutions, at least at low concentrations.^40–46^

In particular, aqueous solutions of perchlorates have received significant attention due to their potential role in explaining the possible recent radar detection of subglacial liquid water below the south pole of Mars.^47,48^

Recent MD simulations have shown that in aqueous solutions of sodium and magnesium perchlorate,^44,45^ an LLCP can be found, as well as water anomalies such as the temperature of maximum density (TMD) curve, the temperature of minimum density (TmD) curve, and maxima in the isothermal compressibility.

In these studies, we have shown that the anomalous properties of water play a crucial role in these systems. Specifically, we found that aqueous solutions of sodium perchlorate (NaClO_4_) and magnesium perchlorate (Mg­(ClO_4_)_2_), with water modeled using the TIP4P/2005 potential, stabilize the HDL-like region of water upon increasing the concentration of the solutes, with a contraction of the LDL-like region, where nucleation is more likely to occur.^1^

Experimental studies have shown that aqueous solutions of perchlorates exhibit eutectic points at remarkably low temperatures, such as 216 K^49^ for magnesium perchlorate and 198.5 K^50^ for calcium perchlorate (Ca­(ClO_4_)_2_), allowing further supercooling for both down to approximately 150 K.^51^ This is relevant as temperatures in Mars’ South Polar Layered Deposits have been recently estimated to be in the range of 171–187 K.^52^

Of all the possible salts that can be dissolved in water, calcium perchlorate stands as the most important from the point of view of the possibility of having liquid water on Mars.

Calcium perchlorate appears in fact to have a stronger tendency than other Martian salts to prevent water from crystallizing during supercooling down to the glass transition and reheating.^51^

Moreover, calcium perchlorate is known for its pivotal role on Mars in relation to brines because of its high hygroscopicity.^53^

Experiments, furthermore, show that even at high concentrations, the hydrogen-bonded network of water is only partially destroyed in the presence of perchlorate ions,^54–56^ and in dilute sodium perchlorate solutions, water anomalies persist up to 2M.^57^

This paper is structured as follows: in section 2, we describe the methodology used to construct the simulation systems; section 3 presents the phase diagrams of Ca­(ClO_4_)_2_ solutions and a comparison with Mg­(ClO_4_)_2_, while section 4 reports the structural results obtained from radial distribution functions (RDFs) of Ca­(ClO_4_)_2_. Finally, section 5 summarizes the key findings and presents the conclusions of this study.

In this article, we study the phase diagram and the structure of supercooled aqueous solutions of Ca­(ClO_4_)_2_ with MD simulations. We also compare the results with those of Mg­(ClO_4_)_2_,^45^ another very common compound on Mars, in order to assess the difference in their behavior.

## 2 Methods

Using the open source software suite GROMACS 5.1.4,^59–61^ we performed MD simulations in the canonical ensemble employing the v-rescale thermal coupling^62^ at concentrations of *C*_1_ = 3.15 wt% and *C*_2_ = 25.7 wt%. We note that these concentrations are well below the experimental solubility at 298 K (≈ 65.3 wt%);^63^ to the best of our knowledge, however, there is no available data for the solubility limit at the lower temperatures relevant to this work. The details of the two solutions are presented in Table 1.

**Table 1:** Concentrations of the Two Systems Investigated Reported in Both Weight Percentage and Molality

	*N*_Ca(ClO_4_)_2__	*N*_H_2_O_	conc. (wt%)	molality (mol/kg)
*C*_1_	10	4072	3.15	0.136
*C*_2_	100	3838	25.7	1.45

We employed the TIP4P/2005 ^19^ to model water and the potential defined in ref 64 to simulate calcium perchlorate.

We used a Lennard–Jones cutoff radius of 0.95 nm and the Lorentz–Berthelot combination rules. Long-range dispersion corrections for the energy and pressure were applied.

We chose a simulation time step of 1 fs and applied periodic boundary conditions. Electrostatics were taken into account by considering the Particle–Mesh Ewald method.^65^

We simulated 26 isochores at concentration *C*_1_ with densities spanning between 890 and 1140 kg/m^3^, and 22 isochores at concentration *C*_2_ with densities spanning between 990 and 1200 kg/m^3^. The isochore at the lowest density investigated coincided for both concentrations with the liquid–gas limit of mechanical stability (LG-LMS), at which cavitation occurred, i.e. vapor bubbles were formed with a consequent sudden rise of the system pressure. For each isochore, we simulated state points at temperatures ranging from 400 to 170 K, for a total number of 1574 simulated state points. The simulation times, for both the equilibration and the production runs, were gradually increased from 100 ps for the state points at a temperature of 400 K to 140 ns for the state points at 170 K.

An image of a 25.7 wt% solution of calcium perchlorate in water is displayed in Figure 1.

**Figure 1:**
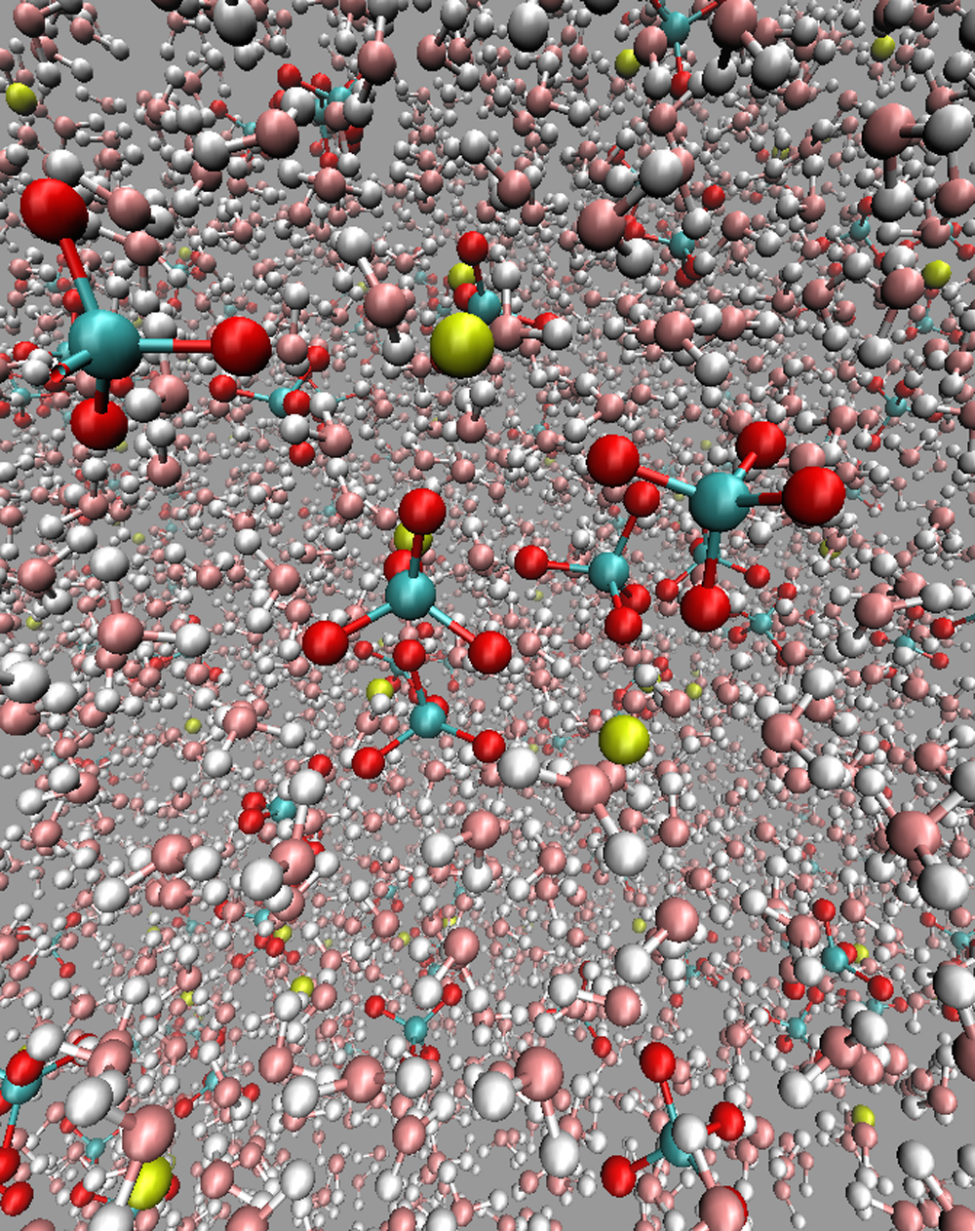
Image of a 25.7 wt% aqueous solution of calcium perchlorate at ρ = 1200 kg/m^3^ and *T* = 200 K. The water molecule is shown in pink (oxygen) and gray (hydrogen), the calcium ion in yellow, and the perchlorate ion in teal (chlorine) and red (oxygens). Created using VMD.^58^

## 3 Thermodynamic Results

In this section, we discuss the phase diagrams of the two solutions, as computed by our simulations.

### 3.1 Results for the *C*_1_ Solution

Figure 2 displays the equation of state for the *C*_1_ solution in the isochore plane, spanning densities from 890 to 1140 kg/m^3^.

**Figure 2:**
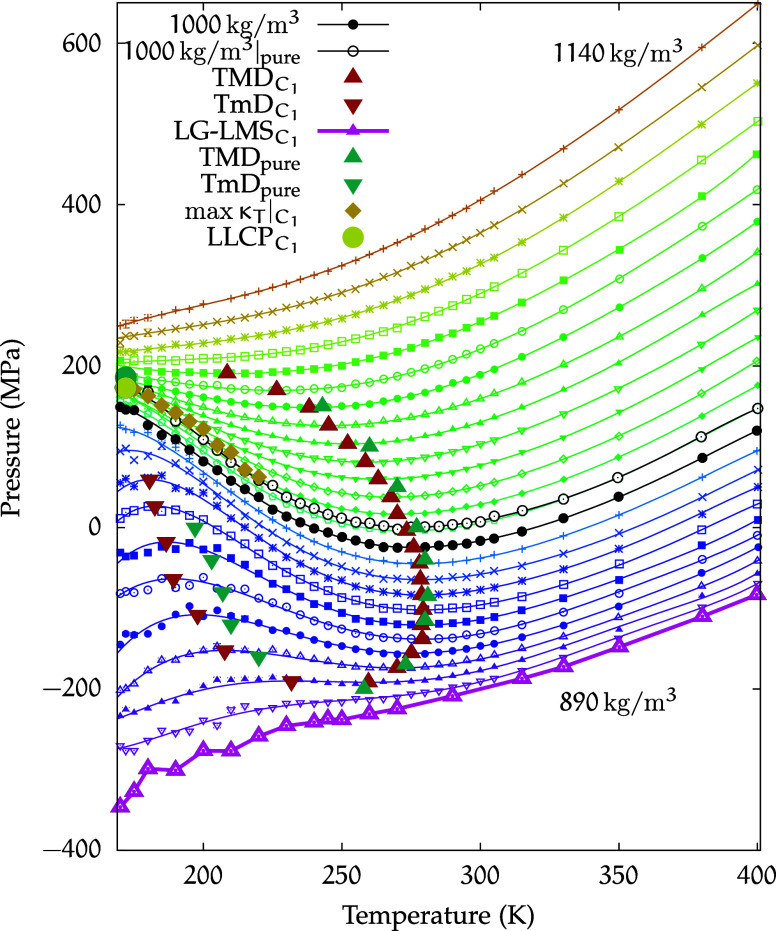
Equation of state for the *C*_1_ solution. Isochores at densities in the range of ρ = 890–1140 kg/m^3^, with increments of Δρ = 10 kg/m^3^, are shown in the (*p*, *T*) plane for temperatures in the range of 400–170 K. Symbols represent simulated state points, while lines correspond to polynomial fits of these points. The TMD curve for the solution is shown as red, filled, upward triangles, while the downward triangles represent the TmD curve. The same convention is used for the teal, filled triangles representing the pure water extrema.^69^ The LLCP is located at *T*_C_ = 172 K and *p*_C_ = 173 MPa (yellow circle). The LLCP of pure water (from ref 21) is indicated by a teal, filled circle, while the Widom line of the *C*_1_ solution is marked by dark yellow, filled diamonds. The LG-LMS isochore at 890 kg/m^3^ is shown as a thicker magenta line at the bottom. Additionally, the isochores for 1000 kg/m^3^ are highlighted for both the *C*_1_ solution (black circles) and pure water (black empty circles).

From Figure 2, we can observe most of the isochores exhibiting a minimum in the range of temperatures that we examine. From these minima, it is possible to calculate the TMD curve, as the isochores satisfy the following condition:

$$\frac{\partial \rho }{\partial T}{|}_{p}=-\frac{\partial p}{\partial \rho }{|}_{T}^{-1}\frac{\partial p}{\partial T}{|}_{\rho }.$$ 1

As per eq 1, we can also determine the temperature of minimum density (TmD) curve from the maxima of the isochores. The locus of points thus determined identifies the *region of density anomaly*, where the density of water decreases upon isobaric cooling and which constitutes the best known among the anomalies of water. At temperatures below the TmD, the regular behavior of simple liquids is restored.^66–68^

Figure 2 also displays the extrema of this region for pure water as known from the literature.^69^

Compared with pure water, a small downward shift in temperature for the TMD curve for the *C*_1_ solution can be observed.

In order to further assess the difference in behavior of the *C*_1_ solution with respect to pure water, highlighted in black in Figure 2 are also the 1000 kg/m^3^ isochores for both the pure phase and the *C*_1_ solution. The isochore of the solution appears to be slightly shifted downward in pressure, while retaining a curvature very similar to that of pure water.

The 890 kg/m^3^ isochore reported in the figure corresponds to LG-LMS, identified as described in section 2.

A second-order critical point between two condensed phases can be identified (see ref 6) by the condition

$$\frac{\partial p}{\partial \rho }{|}_{T_{C}}=0,$$ 2

which indicates the occurrence of a flex point with a horizontal tangent in the isotherm plane.

Equation 2 can be discretised as

$$\frac{p\left(T_{C},\rho \right)}{\Delta \rho }=\frac{p\left(T_{C},\rho +\Delta \rho \right)}{\Delta \rho },$$ 3

implying that the convergence of the isochores occurring at the highest temperature in the (*p*, *T*) plane identifies the onset of the critical region.

In the (*p*, *T*) plane of the phase diagram shown in Figure 2, the isochores appear to converge and the corresponding state points provide an estimate for the location of the critical region at approximately *T* ≈ 172 K and ρ ≈ 1050 kg/m^3^.

This estimate is further supported in the (*p*, ρ) plane (Figure 3) by the horizontal flattening of the isotherms around the flex point, which at *T* = 172 K occurs at ρ = 1051 kg/m^3^.

**Figure 3:**
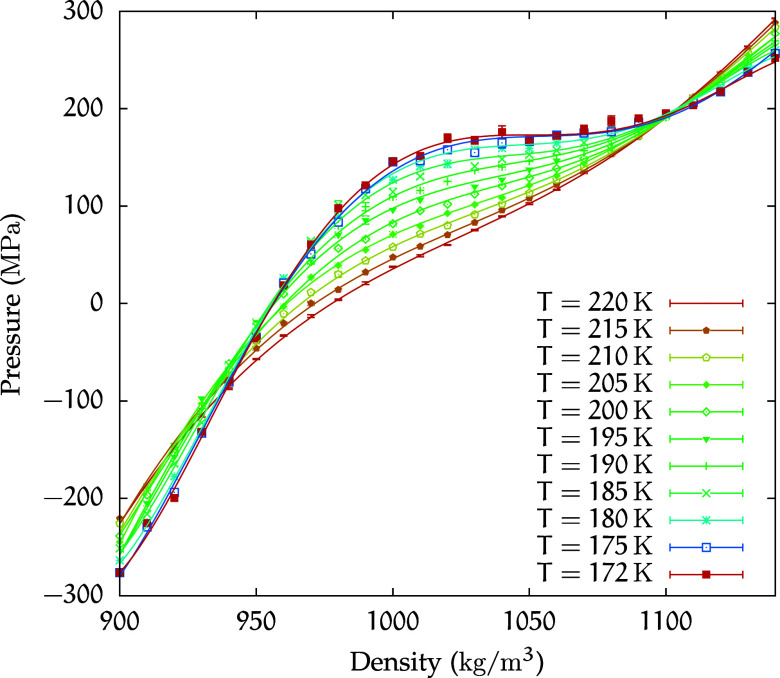
Equation of state for the *C*_1_ solution is plotted in the isotherm plane. The 172 K isotherm curve flattens at 1051 kg/m^3^ and 173 MPa.

As mentioned in the previous section, a second-order critical point is preceded by precursor phenomena arising from the presence of a Widom line. For this reason, thermodynamic response functions exhibit singular behaviors as *T* → *T*_C_.

In Figure 4, we show the behavior of the isothermal compressibility

$$\kappa_{T}=-\frac{1}{V}\frac{\partial V}{\partial p}{|}_{T}$$ 4

at different temperatures as a function of pressure. Points of maximum of κ_*T*_, which provide an estimate of the location of the Widom line near the critical point,^34^ can be observed. Notably, the behavior of κ_*T*_ at 173 MPa confirms the presence of a critical point in the region of density anomaly of this solution.

**Figure 4:**
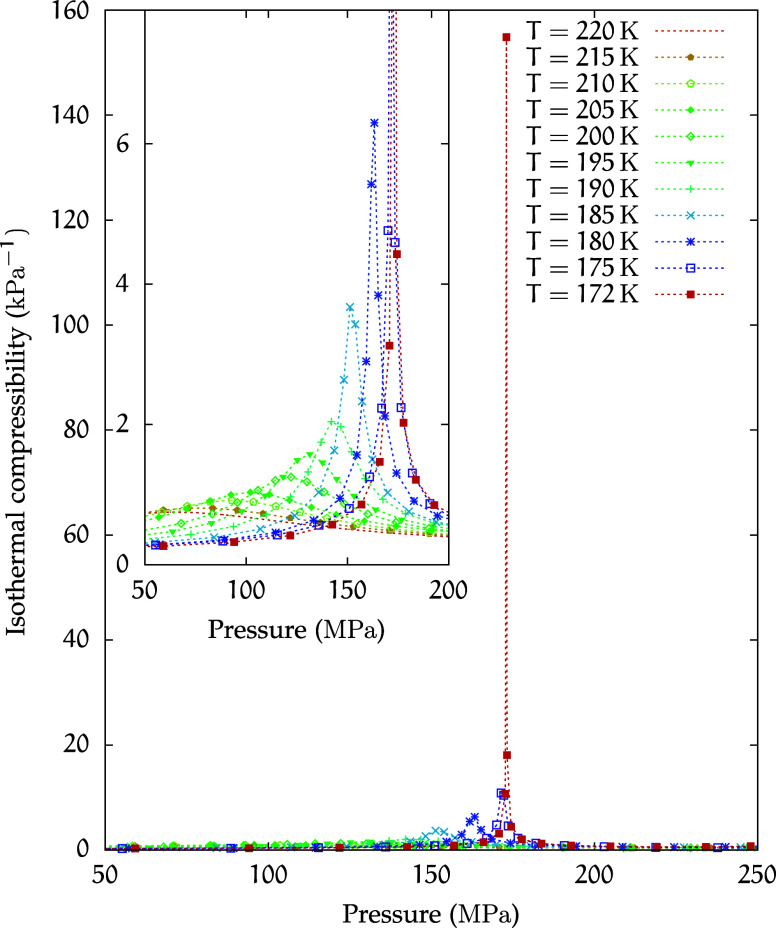
Isothermal compressibility κ_*T*_ for the *C*_1_ solution.

Based on this analysis, we estimate the location of the LLCP at *T* = 172K, ρ = 1051 kg/m^3^, and *p* = 173 MPa.

This point is also reported in Figure 2. For reference, the same figure includes the LLCP of pure TIP4P/2005 water, rigorously determined by Debenedetti et al. in ref 21 at *T*_C_^(pure)^ = 172K and *p*_C_^(pure)^ = 186 MPa.

Compared to pure water, we observe that in the *C*_1_ solution, the LLCP is shifted to a slightly lower pressure, while the critical temperature appears to be the same.

### 3.2 Results for the *C*_2_ Solution

Figure 5 shows the equation of state in the isochore plane for the solution at concentration *C*_2_.

**Figure 5:**
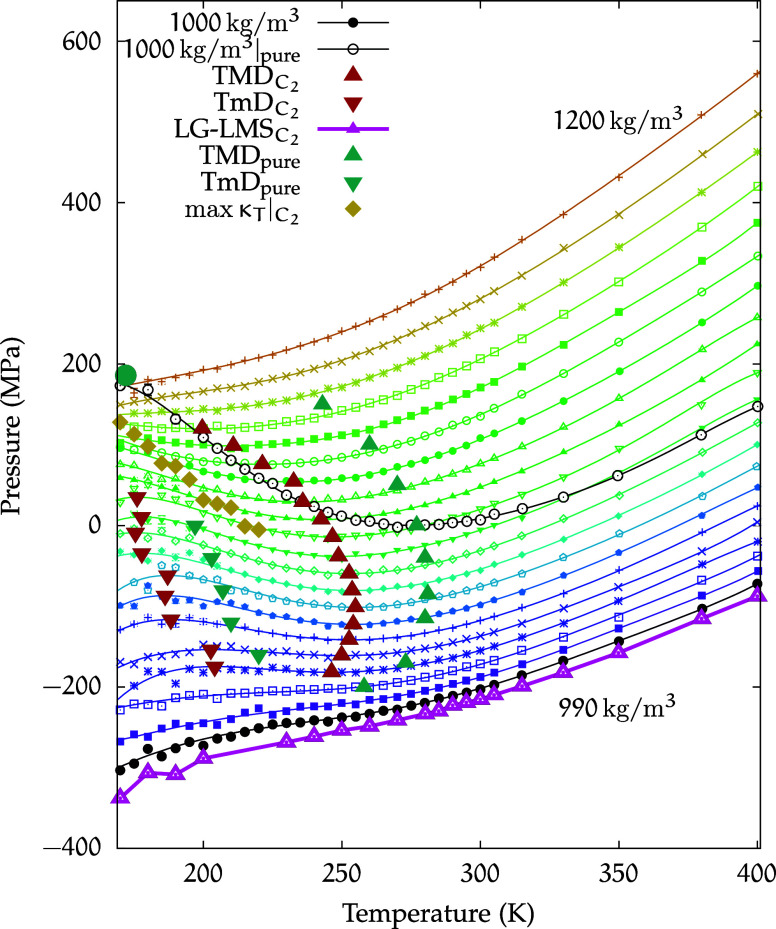
Equation of state for the *C*_2_ solution. Isochores at densities in the range of ρ = 990–1200 kg/m^3^, with increments of Δρ = 10 kg/m^3^, are shown in the (*p*, *T*) plane for temperatures in the range of 400–170 K. Symbols represent simulated state points, while lines correspond to polynomial fits of these points. The TMD curve for the solution is shown as red, filled, upward triangles, while the downward triangles represent the TmD curve. The same convention is used for the teal, filled triangles representing the pure water extrema.^69^ The LLCP of pure water^21^ is indicated by a teal, filled circle, while the isothermal compressibility maxima are marked by dark yellow, filled diamonds. The LG-LMS isochore at 990 kg/m^3^ is shown as a magenta line at the bottom. Additionally, the isochores for 1000 kg/m^3^ are highlighted for both the *C*_2_ solution (black circles) and pure water (black empty circles).

With regard to modifying the thermodynamic behavior relative to pure water, this concentration is considerably high. To quantify this effect, Figure 5 presents the 1000 kg/m^3^ isochores for both the *C*_2_ solution and pure water, similar to the comparison made for the *C*_1_ solution. In this instance, the difference between the pure water isochore and the corresponding *C*_2_ isochore is notable. Furthermore, we observe that all of the *C*_2_ isochores display a much flatter curvature than that of the 1000 kg/m^3^ pure water isochore.

The TMD and TmD curves are still present and for both the *C*_2_ solution and pure TIP4P/2005 water are also depicted in Figure 5. Notably, for the *C*_2_ solution, these curves are shifted toward lower temperatures, while exhibiting no significant shift in pressure.

The LG-LMS was identified as the 990 kg/m^3^ isochore. No significant pressure shift of this line was observed when compared with the *C*_1_ solution.

No evidence of isochore crossing was observed for the *C*_2_ solution, suggesting the absence of critical points within the accessible temperature range. This conclusion is reinforced by the investigation of the behavior of this solution in the isotherm plane, as presented in Figure 6. In contrast to the analogous results for the *C*_1_ solution, no flattening of the isotherms is observed. However, the trend of the isotherms appears to be similar to that of the *C*_1_ solution and pure water. Also, the isothermal compressibility as a function of pressure, shown in Figure 7, reveals that the peak heights increase as temperature decreases, although not, or not yet, so abruptly as for the *C*_1_ solution. Therefore, these observations do not preclude the existence of an LLCP at lower temperatures.

**Figure 6:**
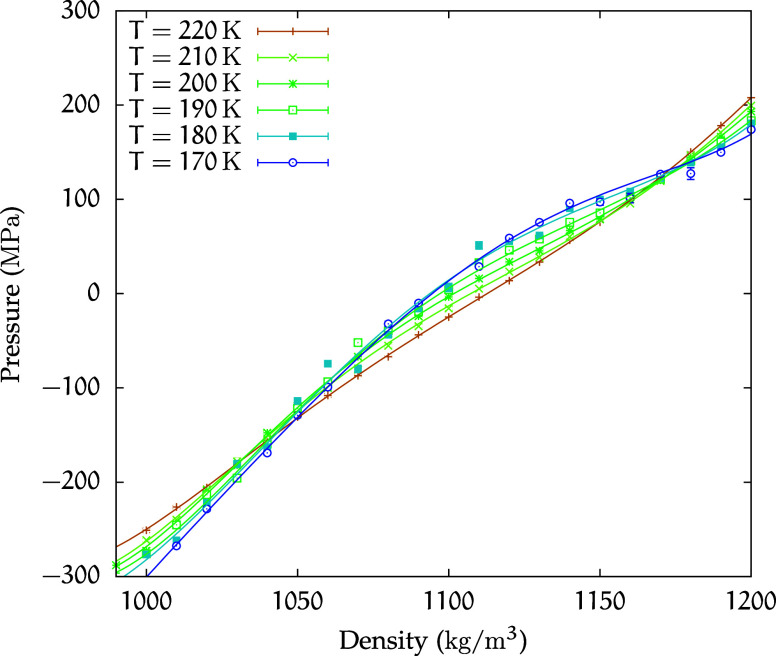
Equation of state in the isotherm plane for the *C*_2_ solution.

**Figure 7:**
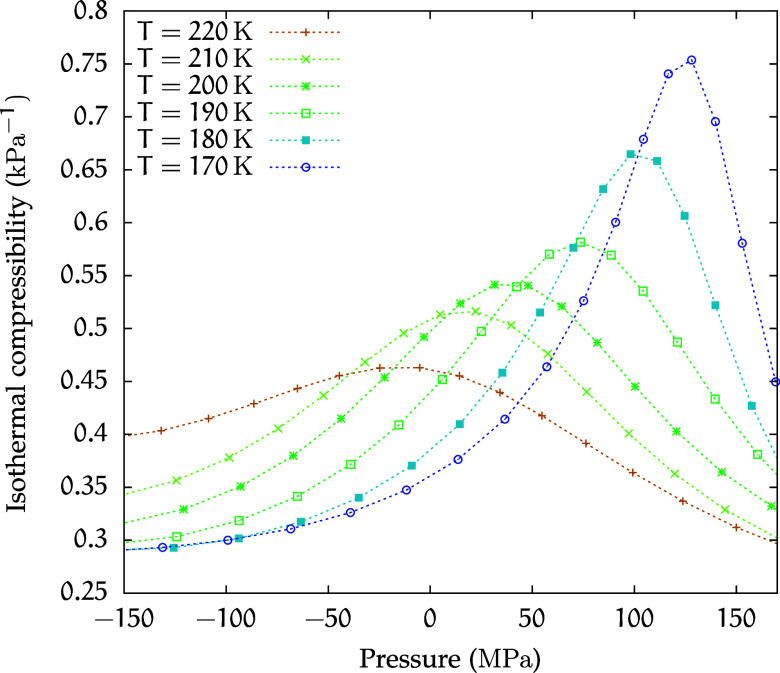
Isothermal compressibility κ_*T*_ for the *C*_2_ solution.

The state points corresponding to the κ_*T*_ maxima are also plotted in Figure 5.

### 3.3 Comparison of the *C*_1_ and *C*_2_ Solutions

A thermodynamic comparison between the two Ca­(ClO_4_)_2_ solutions investigated in this paper is shown in Figure 8, omitting the isochores.

**Figure 8:**
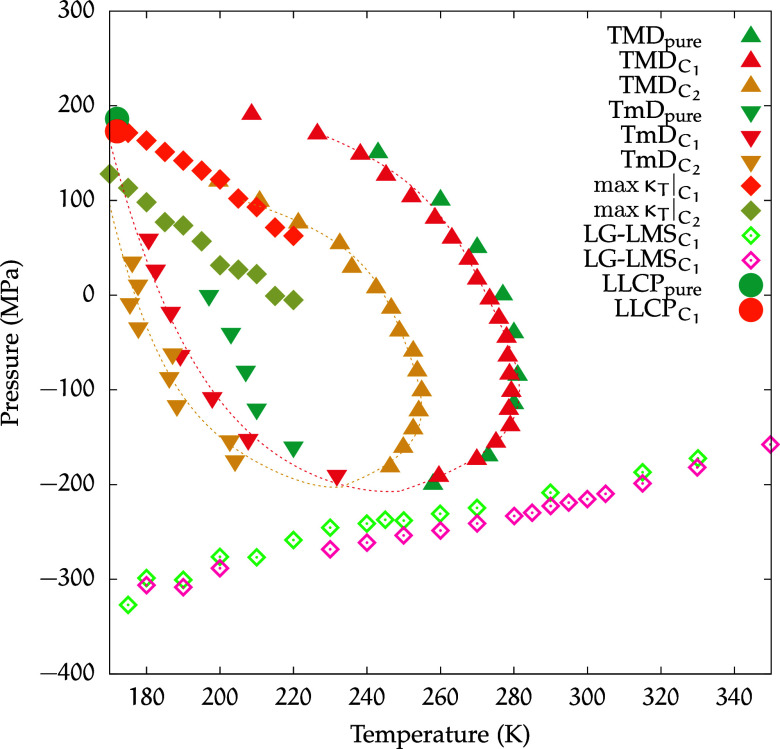
Phase diagram of the two Ca­(ClO_4_)_2_ solutions investigated and pure water, highlighting their water anomalies without the isochores. Dotted lines for the TMDs and the TmDs of the solutions are guides for the eye.

Increasing the solute concentration causes both the TMD and TmD curves to shift toward lower temperatures, although the latter shifts by a smaller amount.

Regarding the critical points, the *C*_1_ solution shows the LLCP shifting slightly lower in pressure compared to pure water, while the critical temperature remains the same. In contrast, the *C*_2_ solution reveals no critical points, at least for the region considered. Furthermore, the LG-LMS displayed no significant pressure variation.

As a result, a contraction of the region of the density anomaly can be observed in Figure 8.

An increase in solute concentration also resulted in a decrease in the pressure at which isothermal compressibility peaks are found, and we remark that these peaks serve as a proxy for the Widom Line, in the one-phase region above the critical point. By definition, being the line of maxima of the correlation length, the Widom Line separates HDL-like state points from LDL-like state points. Even though critical points are not observed in the *C*_2_ solution, its observed isothermal compressibility peaks can still estimate the locations of the strongest HDL–LDL correlation and could be still possibly pointing to an LLCP located at lower temperatures.

A key thermodynamic consequence of increasing salt concentration, illustrated in Figure 9, is therefore the reduction of the LDL-like region upon increasing the concentration of the solutes. This favors vitrification against nucleation, the latter being more favored in LDL. Figure 9 also shows a hypothetical (not computed) location of the HDL LMS, which corresponds to the HDL-to-LDL spinodal in one-component fluids.^6^

**Figure 9:**
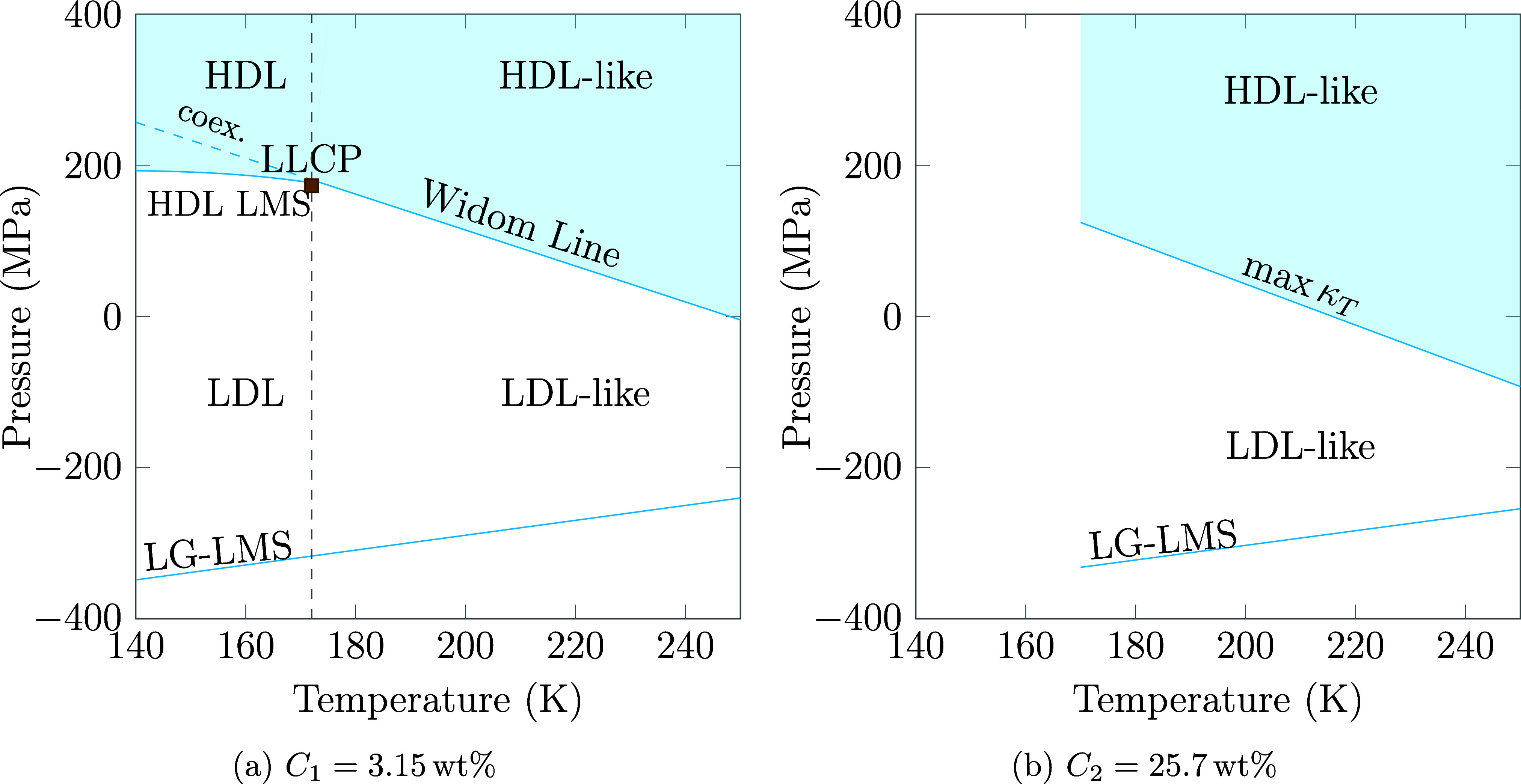
Schematic phase diagrams of the two solutions investigated. (a) Phase diagram for the *C*_1_ solution, including the estimated lines of the HDL LMS below the critical point (not computed) and of coexistence (prolonged from the Widom Line). Below 170 K, the LG-LMS is also prolonged. (b) Shrinkage of the region where LDL fluctuations prevail on the HDL ones in the one-phase region at concentration *C*_2_.

### 3.4 Comparison of the *C*_2_^Mg(ClO_4_)_2_^ and *C*_2_^Ca(ClO_4_)_2_^ Solutions

Because perchlorate solutions are believed to be responsible for the potential detection of liquid water underneath Martian soil,^47,48^ it is important to also assess how the thermodynamic behavior changes when different solutes are considered. For this reason, we will now compare the thermodynamic behavior of the solutions presented in this paper with those of Mg­(ClO_4_)_2_ investigated in ref 45, as the same potential to model the perchlorate ion is shared among them. In particular, the *C*_1_^Ca(ClO_4_)_2_^ and *C*_2_^Ca(ClO_4_)_2_^ concentrations were chosen to precisely match the molality of the *C*_1_^Mg(ClO_4_)_2_^ and the *C*_2_^Mg(ClO_4_)_2_^ solutions, respectively (although their value in wt% is slightly different due to the different masses of Ca and Mg).

The thermodynamic trend shown by these solutes is similar to that of pure water, but while the general thermodynamic behavior for the *C*_1_ solutions is pretty much unchanged with respect to pure water for both magnesium and calcium perchlorate, the thermodynamic effect at concentration *C*_2_ appears to be stronger for calcium perchlorate than for magnesium perchlorate.

Therefore, in Figure 10 we directly compare the phase diagrams of the two different solutions at the highest concentrations investigated along with those of pure water. The figure shows that despite the large shift and shrinkage of the TMD region with respect to that of pure water, this anomaly for water still clearly persists for both magnesium and calcium in aqueous solution with perchlorate. In particular, for calcium, the displacement toward lower temperatures with respect to pure water is more marked than for magnesium.

**Figure 10:**
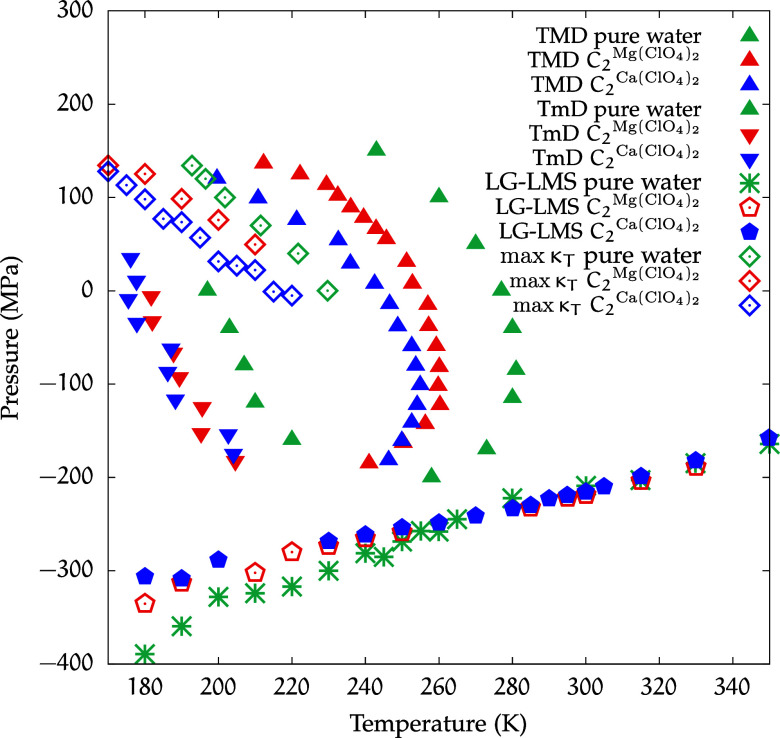
Thermodynamic comparisons for pure water and for aqueous solutions of magnesium perchlorate and calcium perchlorate. The max κ_*T*_ curve for pure water is taken from ref 36.

We further note that calcium, besides inducing a more substantial shrinkage of the density anomaly region, also causes a more substantial shift toward lower pressures of the κ_*T*_ maxima line compared to pure water (points taken from ref 36). We observe that the three LG-LMS lines are always at the same position down to ≈240 K; below this temperature, the pure water LG-LMS lies at lower pressures, and the difference in pressure with respect to the lines of the solutions increases as the temperature further decreases.

This shows that for the supercooled temperatures, both solutions shrink the LDL-like region from below.

Adding this observation to the further shift of the κ_*T*_ maxima line to lower pressures in the case of calcium perchlorate, we see that, in the presence of perchlorate, the Ca is able to shrink more than the Mg in the region below this line, where LDL density fluctuations, which favor nucleation,^1^ prevail over HDL ones.

To better visualize the contraction of the LDL-like region, below the κ_*T*_ maxima line for the two solutions and relative to pure water, we show in Figure 11 a schematic representation of the portion of the phase diagram below and above the κ_*T*_ maxima line, which is a proxy for the Widom line. We can see clearly here that both magnesium and calcium perchlorate shrink the LDL-like region, but calcium perchlorate shrinks it more.

**Figure 11:**
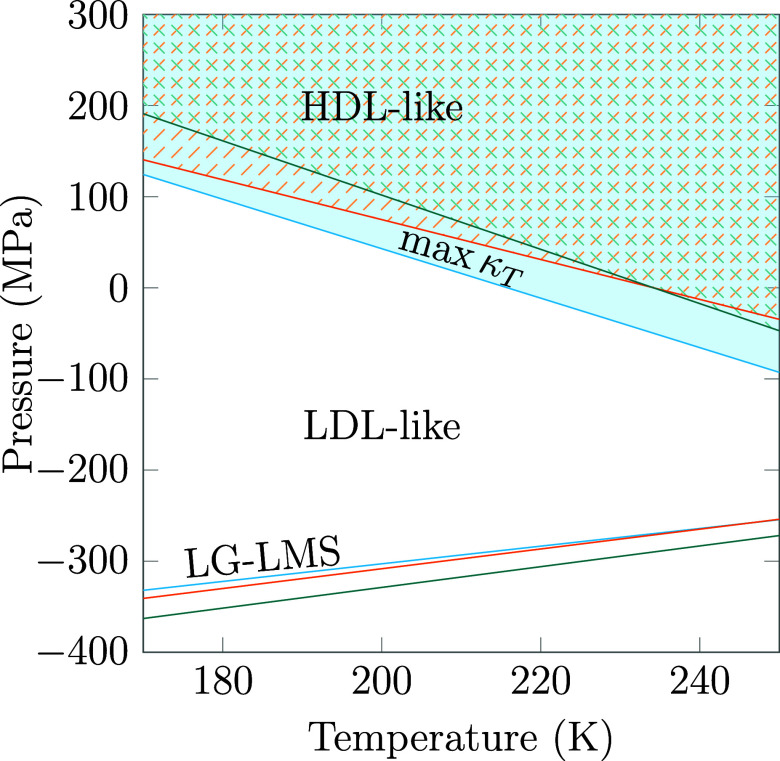
Schematic representation of a comparison of the phase diagrams of *C*_2_^Mg(ClO_4_)_2_^ (in orange, ref 45) and *C*_2_^Ca(ClO_4_)_2_^ (in cyan) solutions and pure water (in teal), showing the stronger effect of calcium perchlorate with respect to magnesium perchlorate. The max κ_*T*_ curve for pure water is taken from ref 36.

These results show that it is possible to have supercooled water on Mars, as calcium perchlorate appears also to play one of the most important roles on the Red Planet in relation to water compared to other minerals because of its high hygroscopicity.^53^

## 4 Structural Results

The structural properties were analyzed using RDFs, *g*(*r*).^6,33^

Because our focus is on understanding the HDL and LDL behaviors of the solutions, we will analyze the RDFs that reveal these features.

We begin our investigation by considering the O_W_–O_W_ RDFs (*g*_O_W_–O_W__(*r*)) for the two solutions and compare them with those of the corresponding pure water system. Given that the isochore curves display significant differences in curvatures and downward pressure shifts with increasing concentration, we selected three sets of densities for comparison across the three systems (pure water, *C*_1_, and *C*_2_). These density triplets, presented in Table 2, were chosen to exhibit comparable thermodynamic behaviors at low temperatures. In particular, we chose isochores with comparable pressures. We note that our analysis allows for a robust comparison among our previous studies on NaClO_4_,^44^ Mg­(ClO_4_)_2_,^45^ and this work on Ca­(ClO_4_)_2_, whereas, for example, an alternative analysis based on number densities would result in the comparison of systems with vastly different mass densities. The criterion for choosing the isochores is applied consistently across all three studies.

**Table 2:** Comparison of Density Triplets for the O_W_–O_W_ RDFs^a^

	ρ_pure_ (kg/m^3^)	ρ_*C*1_ (kg/m^3^)	ρ_*C*__2_ (kg/m^3^)
high-density triplet	1110	1110	1200
medium-density triplet	990	990	1150
low-density triplet	930	930	1060

a Values were selected by matching isochores with similar thermodynamic behaviors at low temperatures.

Studying the *g*_O_W_–O_W__(*r*) makes it possible to discern the structural characteristics associated with HDL-like and LDL-like water. In particular, the LDL phase exhibits enhanced tetrahedral ordering, reflected in a more pronounced second peak and a deeper first minimum relative to the HDL phase.^70–72^

As illustrated in Figure 12 for the three density triplets, all of the systems display HDL characteristics at higher temperatures. Further cooling, as especially evident at 220 and 170 K, leads to a more ordered, LDL-like structure.

**Figure 12:**
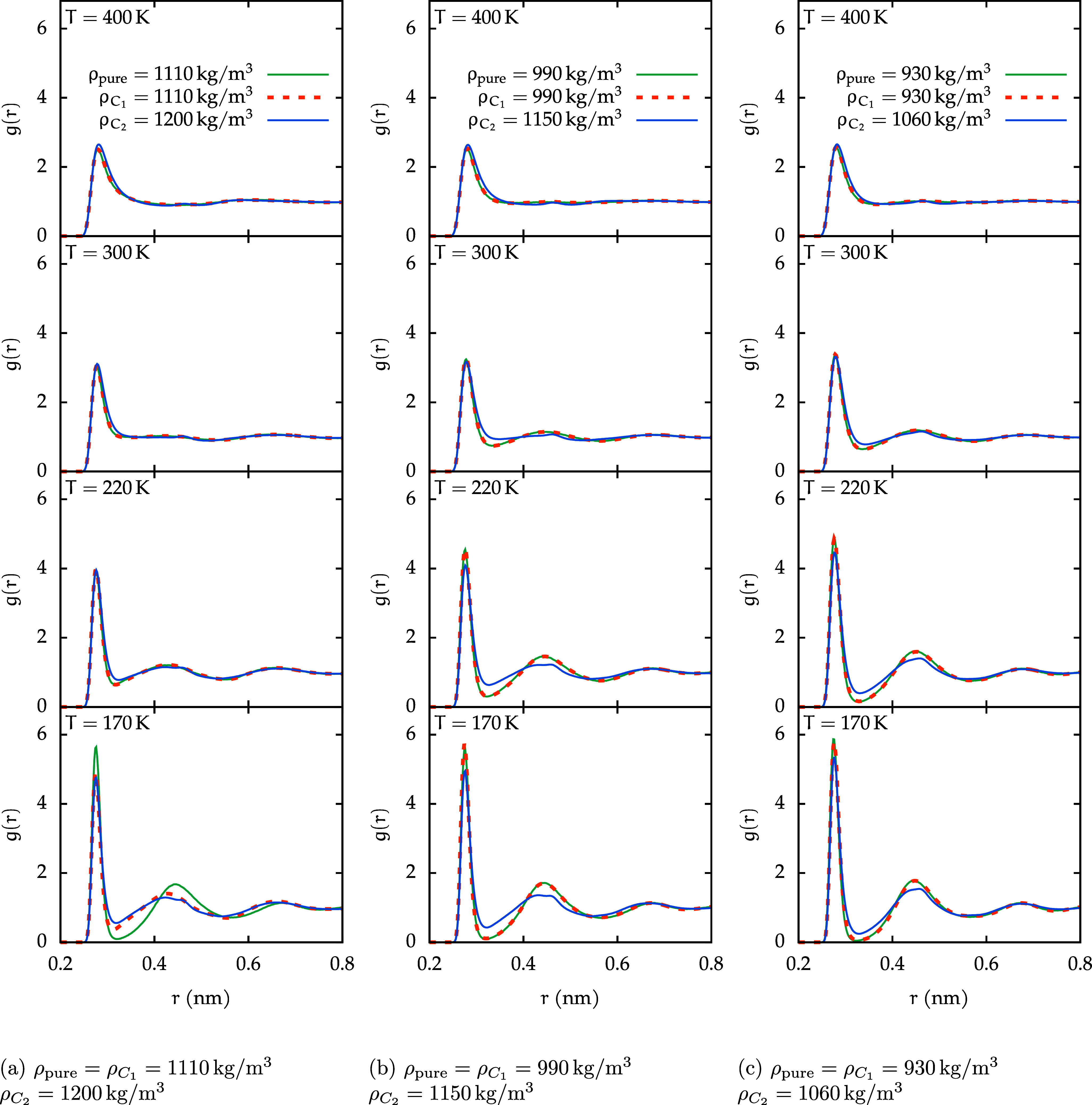
Water–water (O_W_–O_W_) RDFs for pure water, and for the *C*_1_ and the *C*_2_ solutions, corresponding to sets of densities chosen by matching isochore curves with comparable thermodynamics at low temperatures. The RDFs are shown for the three density triplets: (a) high-density, (b) medium-density, and (c) low-density.

This gradual structural shift toward an LDL-like behavior in the one-phase region is clearly observed in pure water across all densities. The *C*_1_ solution closely follows this behavior, with its *g*_O_W_–O_W__(*r*) generally matching that of pure water at corresponding state points. Minor deviations are noticeable, such as at 170 K and 1110 kg/m^3^ (fourth panel in Figure 12a), where pure water is more LDL than the solution.

In contrast, while the *C*_2_ solution follows a similar overall trend, a distinct LDL-like character emerges only at low temperatures and low densities.

In Figure 13 we show the O_W_–O_W_ first shell coordination number, *N*_C_, in order to further characterize the local water structure.

**Figure 13:**
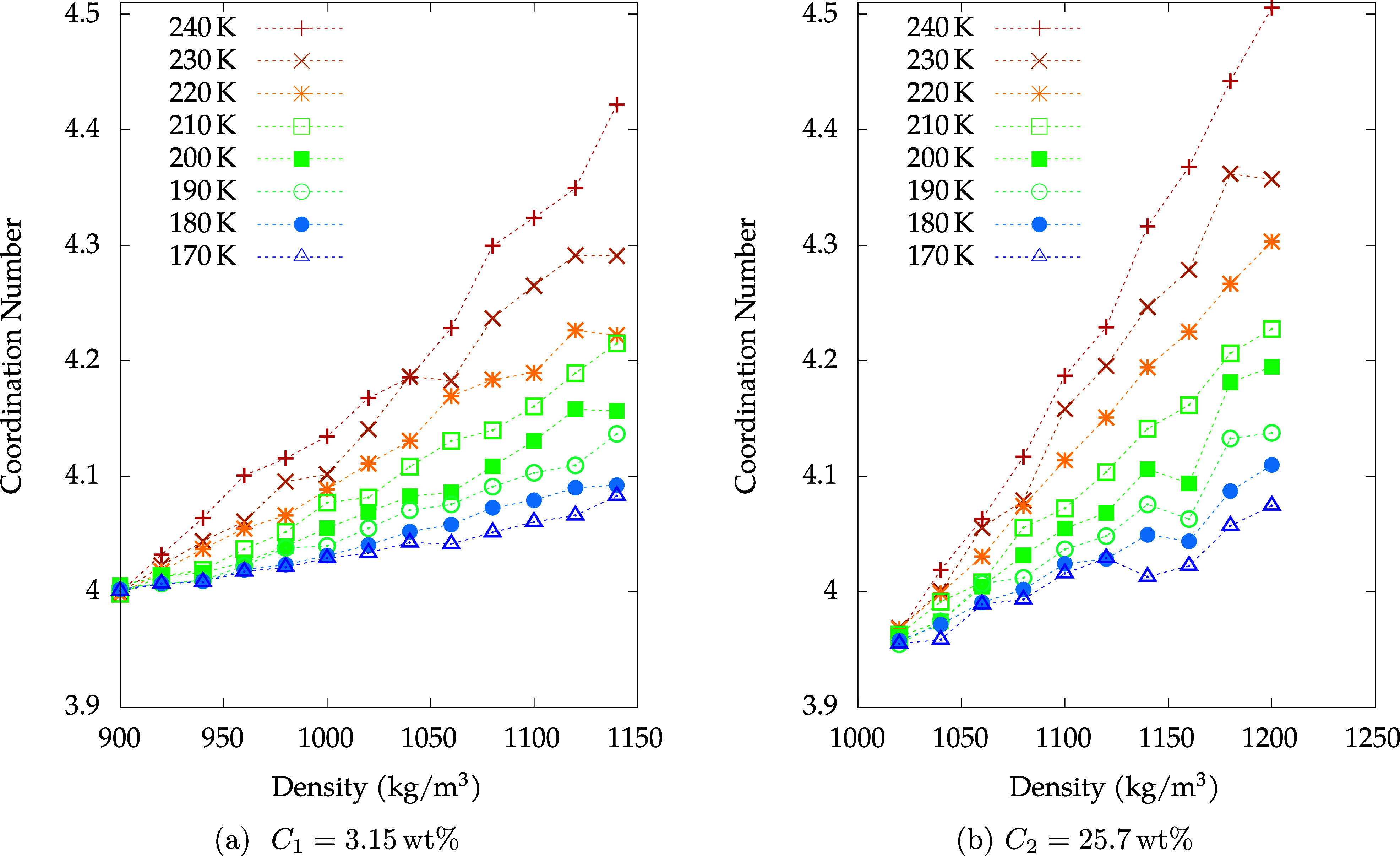
O_W_–O_W_ first shell coordination numbers for the (a) *C*_1_ solution and (b) *C*_2_ solution, plotted as a function of density.

*N*_C_ is defined as follows:

$$N_{C}=4\pi \frac{N_{O_{W}}}{m}\rho \int_{0}^{r_{0}}dr\;g_{O_{W}-O_{W}}(r)r^{2}$$

where *m* is the total mass of the system and *r*_0_ is the location of the O_W_ – O_W_ RDF’s first point of minimum.

While *N*_C_ increases with the density in both solutions, it decreases with increasing concentration at lower densities. This reduction is also attributable to the easier solvation of ions by water molecules as water becomes less LDL, and thus, a more distorted tetrahedral structure emerges. At low densities, low temperatures, and low concentration (*C* = *C*_1_), on the other hand, the coordination number approaches 4, as typical for LDL water.

The RDFs centered on chlorine are shown in Figure 14 for two densities (1200 and 1060 kg/m^3^) and four temperatures (400, 300, 220, and 170 K). These plots illustrate the local ion arrangement around chlorine for hydrogen, calcium, the oxygen of water, and other chlorines.

**Figure 14:**
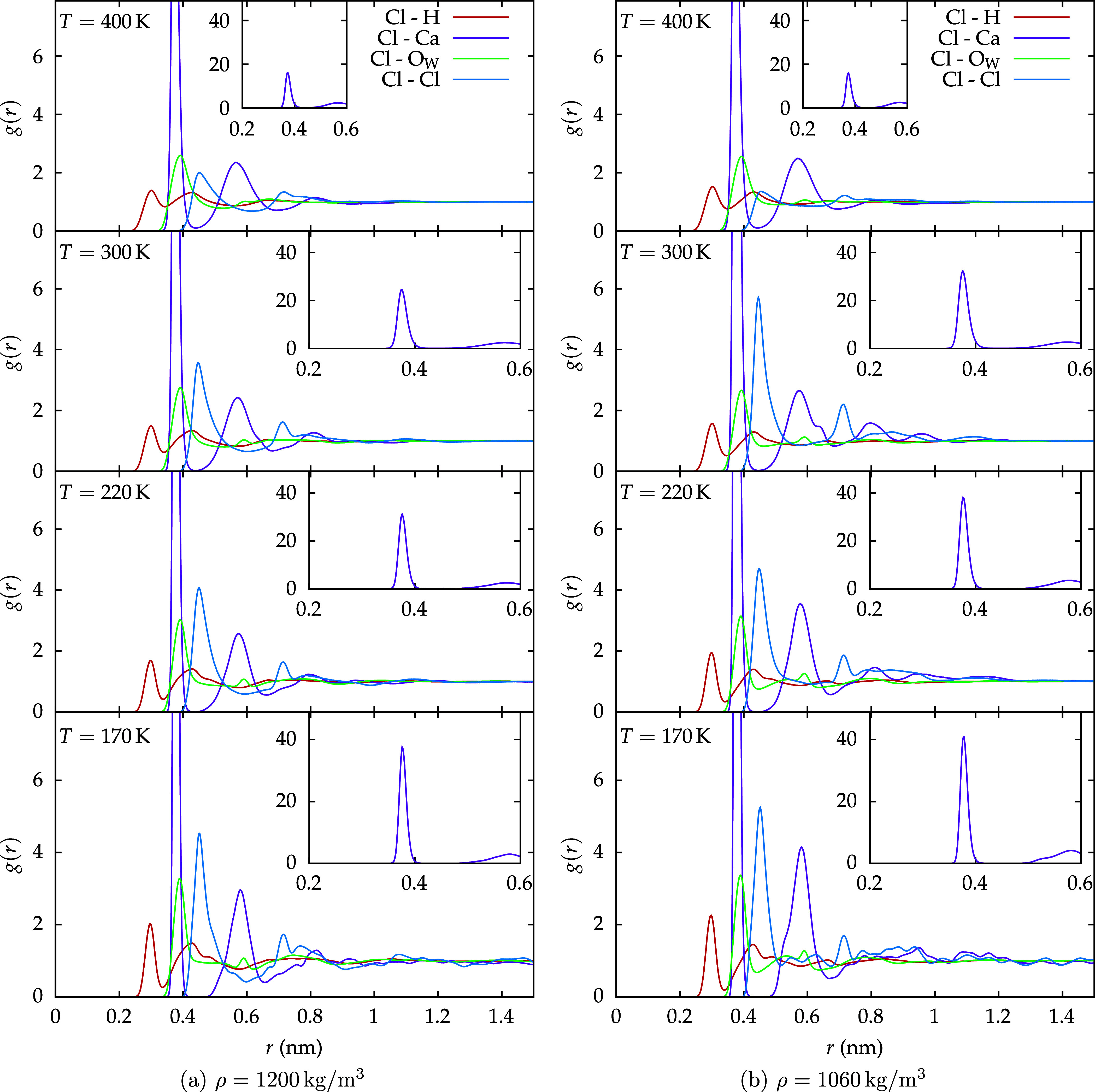
RDFs for Cl–H, Cl–Ca, Cl–O_W_, and Cl–Cl are presented for the *C*_2_ solution at two different densities: (a) ρ = 1200 kg/m^3^ and (b) ρ = 1060 kg/m^3^. The graphs illustrate the coordination of chlorine with hydrogen, calcium, oxygen, and chlorine, respectively. Additionally, the panels highlight the Cl–Ca RDF, showing the height of the first peak on different scales.

The first atom to appear in this arrangement is hydrogen, showing a first peak at around 0.3 nm, indicating that Cl is always surrounded by a cage of water. The shape and height of this peak appears to be substantially density-independent at higher temperatures. Upon decreasing the temperature, the peak becomes more structured and slightly more sensitive to density, as the low-density first peaks at 220 and 170 K are more pronounced than the high-density ones. This behavior is mirrored by the Cl–O_W_ RDF.

The Cl–Cl RDF shows a well-defined first peak at approximately 0.45 nm. At lower temperatures, the peak is slightly higher, and it is narrower when the density is lower.

The RDF for Cl–Ca shows a pronounced peak at around 0.37 nm. This peak appears to become more and more prominent in the LDL-like region of water, especially upon lowering the temperature, as the LDL phase is known to exclude ions.^1^ Comparing these findings to previous results for magnesium perchlorate,^45^ the substantially density-independent Cl–Mg pairing occurred at a shorter distance of approximately 0.34 nm, reflecting the smaller ionic radius of Mg.

Thus, the structural analysis confirms the presence of water anomalies in the solutions, although the water–water RDFs show a general decrease in the LDL character compared to that of pure water.

## 5 Conclusions

In this work, we employed MD simulations to investigate the thermodynamic and structural properties of supercooled aqueous solutions of Ca­(ClO_4_)_2_ at concentrations of 3.15 and 25.7 wt%, using the TIP4P/2005 model for water,^19^ in order to understand how this salt, known for its abundance on Mars and for its remarkably low eutectic temperature,^47,48,51^ modifies the phase diagram of supercooled water.

Thermodynamic analysis confirmed that the characteristic anomalies of water, such as the TMD and TmD curves, persist in both solutions, although they are shifted toward lower temperatures as the solute concentration increases. For the low-concentration solution (*C*_1_), we identified a second-order LLCP at *T*_*C*_ = 172 K and *p*_*C*_ = 173 MPa, indicating the persistence of the transition between the LDL and HDL phases of water. This LLCP is located at a slightly lower pressure compared to that of pure TIP4P/2005 water^21^ but at the same critical temperature.

In the higher-concentration solution (*C*_2_), no LLCP was observed within the explored range of temperatures and pressures. Upon decreasing the temperature, however, the maxima of the isothermal compressibility (κ_*T*_), while not showing divergence, increase, thus not ruling out the possibility of an LLCP existing at temperatures below 170 K. The solute has a pronounced effect at this concentration: a contraction of the region of density anomaly is observed, along with a marked shift of the TMD and TmD lines compared to that of pure water.

Furthermore, importantly, a contraction of the LDL thermodynamic region, where nucleation is favored,^1^ is observed.

Comparison with our previous study^45^ performed using the same potentials^19,64^ on Mg­(ClO_4_)_2_ indicates that, at the same molality, calcium perchlorate exerts a more pronounced thermodynamic effect on the phase diagram of water both in shrinking its LDL-like region and in shrinking and shifting the TMD region to lower temperatures.

From a structural standpoint, analysis of the O_W_–O_W_ RDFs and O_W_–O_W_ coordination numbers revealed that increasing the Ca­(ClO_4_)_2_ concentration favors structural behavior more similar to that of the HDL phase, compared to that of pure water. Nonetheless, the LDL-like behavior is still present at low densities and temperatures, as also reflected by the Cl–Ca ion pairing being more pronounced in these regions, as the LDL structure of water tends to exclude solutes.

Overall, our results indicate that calcium perchlorate exerts the strongest alteration and shift to lower temperatures among perchlorate salts to the phase diagram of supercooled water, significantly contracting the LDL-like region, which is known for favoring ice nucleation.^1^ Indeed, Ca^2+^ has a stronger disordering effect on water, and it might be related to its larger ionic radius relative to that of Mg^2+^, which lowers its charge density. These findings are consistent with the hypothesis that calcium perchlorate solutions can contribute to the presence of subglacial water bodies^48^ in extremely cold environments such as the Martian subsurface.
